# Effectiveness and pregnancy outcomes of ultrasound-indicated and physical examination-indicated cervical cerclage: a retrospective study from a single centre

**DOI:** 10.1186/s12884-024-06659-w

**Published:** 2024-07-08

**Authors:** Linxiang Huang, Wenting Wang, Yuchuan Wang, Jie Chen, Shuping Jin, Xiaoxuan Qi, Yujia Qian, Qing Cheng

**Affiliations:** 1 Department of Obstetrics, Women’s Hospital of Nanjing Medical University, Nanjing Women and Children’s Healthcare Hospital, Nanjing, 210004 China; 2 Department of Gynecology and Obstetrics, The Second Affiliated Hospital of Nantong University, Nantong, 226001 China

**Keywords:** Cervical incompetence, Cervical cerclage, Cerclage indication, Pregnancy outcome, Preterm birth, Twin pregnancy

## Abstract

**Objective:**

Preterm birth (PTB) is the leading cause of neonatal morbidity and mortality worldwide, and cervical incompetence (CIC) is a significant contribution. Cervical cerclage (CC) is an effective obstetric intervention. However, many clinical factors affect the success rate of surgery. The objective was to investigate and compare the pregnancy and neonatal outcomes of patients who underwent ultrasound- and physical examination-indicated cervical cerclage and to explore the influencing factors of preterm delivery before 34 weeks.

**Methods:**

The sociodemographic characteristics and clinical data of patients with a diagnosis of cervical incompetence who underwent ultrasound- and physical examination-indicated transvaginal cervical cerclage at Nanjing Maternal and Child Health Hospital from January 2020 to December 2022 were retrospectively analyzed. The pregnancy and neonatal outcomes of the patients were evaluated. Continuous variables were compared using Student’s t test (for normally distributed data) or the Mann-Whitney U test (for nonnormally distributed data). Categorical variables were analysed using the chi-square test or Fisher’s exact test. Additionally, logistic regression analyses and receiver operating characteristic curves were used to evaluate the associations of inflammatory markers with maternal and neonatal outcomes.

**Results:**

This study included 141 participants who underwent cervical cerclage, including 71 with ultrasound-indicated cerclage and 70 with physical examination-indicated cerclage. Compared to those in the ultrasound-indicated cerclage group, the duration from cerclage to delivery, birth weight, and APGAR score in the physical examination-indicated cerclage group were significantly lower, and the rates of delivery at < 28 weeks, < 32 weeks, < 34 weeks, and < 37 weeks of gestation and neonatal mortality were significantly higher (all *P* < 0.05). Compared to those in the physical ultrasound-indicated cerclage group, in the physical examination-indicated cerclage group, maternal blood inflammatory markers, such as C-reactive protein (CRP), the systemic immune-inflammation index (SII) and the systemic inflammation response index (SIRI) were significantly higher (*P* < 0.05). Additionally, maternal blood inflammatory markers, such as the CRP, white blood cell count, platelet to lymphocyte ratio (PLR), SII, and SIRI were significantly higher in the group with delivery before 34 weeks of gestation. Furthermore, the results demonstrated that twin pregnancy had the highest OR for preterm delivery before 34 weeks of gestation (OR = 3.829; 95% CI 1.413–10.373; *P* = 0.008), as well as the following: the SII level (OR = 1.001; 95% CI 1.000-1.002; *P* = 0.003) and CRP level (OR = 1.083; 95% CI 1.038–1.131; *P* = 0.022). The risk factors for preterm delivery before 34 weeks of gestation were twin gestation, an increased SII level and an increased CRP level, which had good combined predictive value.

**Conclusion:**

In patients with cervical insufficiency, ultrasound-indicated cervical cerclage appears to lead to better pregnancy outcomes than physical examination-indicated cerclage. Twin pregnancy and maternal blood inflammatory markers, such as the CRP level and the SII, are associated with preterm delivery before 34 weeks of gestation.

## Introduction

Preterm birth (PTB) is defined as birth before 37 completed weeks of gestation from the first day of the last menstrual period [1]. It occurs in approximately 12% of pregnancies and is the leading cause of perinatal morbidity and mortality worldwide [2], which is a significant public health problem. Many risk factors have been shown to be related to preterm birth [3]. Among them, cervical incompetence (CIC) is a leading cause of PTB, and the PTB rate among pregnant women with cervical insufficiency is 3.3 times higher than that among pregnant women without cervical insufficiency [4]. CIC, a common clinical challenge in obstetrics, refers to the progressive, painless shortening, flattening, and dilation of the cervix in the absence of uterine contractions before 37 weeks of gestation, leading to irreversible miscarriage or preterm delivery [5]. The general incidence of cervical incompetence has been estimated to be 1% and is higher in patients with a history of second-trimester abortion or preterm birth [6]. Cervical cerclage (CC) is the only effective treatment for cervical incompetence. By using surgical stitches or cerclage straps, cervical cerclage not only provides a certain degree of structural support for patients with cervical incompetence but also maintains the length of the cervix and the endocervical mucus plug as a mechanical barrier against retrograde infection [7]. CC is classified as history-indicated, ultrasound-indicated, and physical examination-indicated cerclage [8]. CC can increase gestational age (GA) and reduce the risk of preterm birth caused by cervical insufficiency [9, 10]. However, many clinical factors affect the success rate of the surgery. Many studies have reported that maternal blood inflammatory markers such as the C-reactive protein (CRP) level, white blood cell (WBC) count, neutrophil-lymphocyte ratio (NLR), platelet to lymphocyte ratio (PLR), the systemic immune-inflammation index (SII), and the systemic inflammation response index (SIRI) are associated with preterm birth and adverse pregnancy outcomes. The SII and SIRI, as emerging inflammatory markers, integrate various subgroups of white blood cells and reflect the local immune status and systemic inflammation of the entire human body, which can be calculated using simple formulas from blood routine indicators [11]. Through retrospective analysis of the clinical data of pregnant women who were diagnosed with cervical incompetence and underwent ultrasound- and physical examination-indicated transvaginal cervical cerclage, the study aimed to evaluate and compare the effectiveness and pregnancy outcomes of the two groups. We further explored the relationship of maternal blood inflammatory markers and other factors with preterm delivery before 34 weeks of gestation.

## Materials and methods

### Participants

The study retrospectively included 141 obstetric patients with cervical incompetence who underwent transvaginal cervical cerclage at Nanjing Maternal and Child Health Hospital from January 2020 to December 2022. Of these, 71 (50.35%) had ultrasound-indicated cerclage, while 70 (49.65%) had physical examination-indicated cerclage. The ultrasound-indicated cerclage group in this study included patients with a cervical length (CL) ≤ 25 mm or V-shaped or U-shaped notches as detected by mid-trimester transvaginal ultrasound. The group of patients with physical examination-indicated cerclage, also called rescue or emergency cerclage, included patients with painless, progressive dilatation of the cervix with or without membrane bulging through the external cervical os detected on vaginal examination. All patients included in the study signed informed consent forms.

### Inclusion and exclusion criteria

The inclusion criteria were as follows: (1) > 18 years old, (2) gestational weeks 12–28, (3) no severe complications, and (4) delivery in our hospital and complete data. The exclusion criteria were as follows: (1) active uterine bleeding, (2) fetal malformations or stillbirth, (3) regular uterine contractions, (4) severe infection, (5) comorbid serious medical and surgical diseases not suitable for continuing pregnancy, and (6) incomplete clinical data.

### Observation indicators

The following clinical information of patients were collected: (1) General situation and high risk factors: age, body mass index (BMI) (at cervical cerclage), single/twin pregnancy, reproductive technology, previous history of adverse pregnancy outcomes, gravidity, parity, recurrent miscarriage (RM), recurrent pregnancy loss (RPL), history of hysteroscopic surgery and history of cervical surgery; (2) Clinical data of the present cervical cerclage surgery: gestational week at cervical cerclage, reproductive tract infection (RTI), CRP levels and routine blood test results such as the SII (Platelet count×neutrophil count/lymphocyte count) and SIRI (monocyte count×neutrophil count/lymphocyte count) before and after cerclage (maximum value 1–3 days after surgery) and comorbid medical conditions such as gestational diabetes mellitus (GDM) and hypertension disorders of pregnancy (HDP); (3) Maternal and foetal outcomes: gestational week, extended days, mode of delivery (spontaneous labour or caesarean section), full-term delivery, delivery at < 37 weeks, < 34 weeks, < 32 weeks, and < 28 weeks of gestation, neonatal survival, birth weight, APGAR score and complications such as postpartum haemorrhage (blood loss of more than 500 ml after vaginal delivery or more than 1000 ml after caesarean delivery).

### Surgical method

McDonald’s cerclage was adopted at the time of diagnosis for the ultrasound- indicated or physical examination-indicated group. The patient was placed in the bladder lithotomy position, and transvaginal cervical cerclage was performed under combined lumbar-epidural anesthesia. After routine disinfection of the vulva and vagina, a sterile sheet was laid, and a catheter emptied the bladder. Vaginal hooks were used to expose the cervix, check the condition of the cervix, and disinfect the area around the cervix again. The standardized transvaginal McDonald’s cerclage procedure was performed with Mersilene tape (Polyester Fiber Suture) in all cases. The sutures were inserted into the anterior, suitable lateral cervical muscle layer. They left lateral parts, placed around the cervix in a purse-string fashion and firmly tied anteriorly. The knot was tightened, and the tail of the thread was retained at approximately 3 cm. For patients with an amniotic sac protruding from the cervical canal, the amniotic sac was retracted with a water sac, and the knot was closed to the cervical canal. Two pieces of iodophor gauze were placed inside the vagina for 24 h to promote hemostasis. All patients received prophylactic antibiotic therapy once preoperatively and postoperatively. Postprocedural progesterone support was not given. In the event of regular contractions, premature repair of membranes, severe infection, or other emergencies, the stitches were removed promptly. If there were no exceptional circumstances, the cerclage sutures were removed before delivery or at 36–37 weeks of pregnancy.

### Statistical analysis

The Statistical Package for the Social Sciences (SPSS) version 27.0 software was used for statistical analysis of the research data. The normality distribution of measurement data was tested by the Shapiro-Wilk test. For parameters with a normal distribution, $\stackrel{-}{x}$ ± s were reported, and a t test was used for the mean value between groups with homogeneous variance. If the variances were heterogeneous, the Mann-Whitney U test was used, and the data were represented by the median (interquartile interval) and M (P25, P75). Count data were analyzed by the chi-square test or Fisher’s exact test, and the data are given as n (%). The Kaplan‒Meier curve was used for the survival analysis of gestational latency. *P* < 0.05 indicated that the difference was statistically significant. Additionally, the gestational age at delivery was divided into ≥ 34 weeks and < 34 weeks for univariate analysis, and then stepwise discriminant analysis was performed to screen variables. Factors with *P* < 0.05 were analyzed by multivariate logistic regression to compare the influencing factors of the differences between the two groups. The area under the receiver operating characteristic curve (ROC) was used to evaluate the efficiency of twin pregnancy and inflammatory markers (the CRP level and SII) in predicting preterm delivery before 34 weeks of gestation. The results are expressed as odds ratios (ORs), 95% confidence intervals (CIs), and *P* values. In all statistical tests, differences were considered statistically significant at *P* < 0.05.

## Results

### Demographic information

A total of 141 patients were included in the study, of whom 71 patients were in the ultrasound-indicated cervical cerclage group, and 70 patients were in the physical examination-indicated cervical cerclage group. The mean age was 31.11 ± 3.77 years old, and the mean BMI at cervical cerclage was 25.50 ± 3.26 (cm^2^ / kg). Among the patients, 30 (21.28%) had twin pregnancies, while the others had singleton pregnancies. Of the patients with singleton pregnancies, 46 (32.62%) underwent assisted reproductive technology (ART) treatment. There were no significant differences in many baseline characteristics, such as age, BMI, number of pregnancies, conception method, gravidity, parity, history of recurrent abortion, history of RM, history of hysteroscopic surgery, and history of cervical conization, between the two groups (*P* > 0.05). The difference in the history of recurrent pregnancy loss between the two groups was statistically significant (*P* < 0.05). Apart from the RPL factor, the demographic and historical information of the two groups of participants were similar (Table 1).

**Table 1:** Comparison of demographic and historical information of patients

	Overall (*n* = 141)	Ultrasound-indicated cerclage (*n* = 71)	Physical examination-indicated cerclage (*n* = 70)	Statistic	*P* value
Age (years, $\stackrel{-}{x}$ ± s)	31.11 ± 3.77	31.15 ± 3.77	31.12 ± 3.80	T = 0.131	0.896
Advanced maternal age (n, %)	30 (21.28%)	15 (14.08%)	15 (21.43%)	X^2^ = 0.002	0.965
BMI (kg / m^2^)	25.50 ± 3.26	25.16 ± 3.07	25.82 ± 3.44	T = -1.231	0.220
Singleton / twin pregnancy				X^2^ = 0.002	0.965
Singleton pregnancies (n, %)	111 (78.72%)	56 (78.97%)	55 (78.57%)		
Twin pregnancy (n, %)	30 (21.28%)	15 (21.13%)	15 (21.43%)		
Natural conception / ART				X^2^ = 0.175	0.676
Natural conception (n, %)	95 (67.38%)	49 (69.01%)	46 (65.71%)		
ART	46 (32.62%)	22(30.99%)	24 (34.29%)		
Gravidity (times)	2 (1–3)	2 (1–3)	2 (1–3)	Z = -1.884	0.060
Parity (times)	0 (0–1)	0 (0–1)	0 (0–1)	Z = -0.528	0.5598
RM (n, %)	6 (4.26%)	5(7.04%)	1 (1.42%)	-	0.209
RPL (n, %)	13 (9.20%)	10 (14.08%)	3 (4.29%)	X^2^ = 4.044	0.044
History of preterm delivery (n, %)	16 (11.35%)	10 (14.1%)	7 (10.0%)	X^2^ = 0.555	0.456
History of hysteroscopic surgery (n, %)	29 (20.57%)	17 (23.94%)	12 (17.14%)	X^2^ = 0.998	0.318
History of cervical conization (n, %)	3 (2.13%)	0 (0%)	3 (4.29%)	-	0.120

Data are presented as numbers (percentages), means ± standard deviations or medians (interquartile ranges). Continuous data were analysed with t tests (for normally distributed data) and expressed as *t* or were analysed with the Mann-Whitney U test (for nonnormally distributed data) and expressed as *Z*. Categorical data were compared using chi-squared tests or Fisher’s exact test and are expressed as *X*^*2*^. - Indicates that Fisher’s exact probability method was used, and no chi-square value was output

### Clinical characteristics

The serological parameters and clinical characteristics of the current pregnancy are presented in Table 2. There was no significant difference between the two groups in terms of the WBC counts, lymphocyte counts, or monocyte counts. However, the CRP level, neutrophil count, NLR, PLR, SII, and SIRI of the physical examination-indicated cervical cerclage group were higher than those of the ultrasound-indicated group. The differences were significant (*P* < 0.05). The incidence rates of clinical complications such as thyroid dysfunction and hyperlipidaemia in the ultrasound-indicated group were 25.4% and 15.5%, respectively, which were higher than those in the physical examination-indicated cerclage group (10% and 2.9%) (*P* < 0.05). There were no differences in the rates of RTI, gestational diabetes, or gestational hypertension between the two groups.

**Table 2:** Serological parameters and clinical characteristics of the patients

	Overall (*n* = 141)	Ultrasound-indicated cerclage (*n* = 71)	Physical examination-indicated cerclage (*n* = 70)	Statistic	*P* value
Serum parameters					
CRP(10^9^ / L)	10.06 (5.53–19.18)	8.39 (3.92–15.25)	14.54 (7.51–23.51)	Z = 3.589	< 0.001
WBCs (10^9^ / L)	10.09 ± 1.96	9.91 ± 2.14	10.28 ± 1.75	T = 1.125	0.263
Neutrophils (10^9^ / L)	7.89 (6.82–9.03)	7.36 (6.29–8.91)	8.22 (7.13–9.22)	Z = 2.099	0.036
Lymphocytes (10^9^ / L)	1.58 (1.30–1.93)	1.58 (1.32–1.93)	1.54 (1.28–1.91)	Z = 0.394	0.694
Platelets (10^9^ / L)	226.53 ± 50.61	220.65 ± 52.07	232.50 ± 48.74	Z = -1.395	0.165
NLR	1.96(1.53–2.73)	2.08 (1.64–3.36)	1.92 (1.48–2.35)	Z = 2.561	0.010
PLR	138.89 (112.09–171.67)	128.28 (105.83–164.19)	143.65 (122.66–174.23)	T = 1.897	0.058
SII (10^9^ / L)	1065.60 (829.74–1430.002.41)	938.39 (754.38–1350.95)	1143.51 (917.76–1486.57)	Z = -3.229	0.001
SIRI (10^9^ / L)	2.41 (1.79–3.28)	2.04 (1.49–3.18)	2.59 (2.02–3.31)	Z = -2.635	0.008
RTI (n, %)	42 (29.79%)	20 (28.17%)	23 (32.86%)	X^2^ = 0.006	0.937
Clinical comorbidities (n, %)					
Anaemia (n, %)	72 (51.06%)	38 (53.52%)	34 (48.57%)	X^2^ = 0.346	0.557
GDM (n, %)	43 (30.50%)	27 (38.03%)	16 (22.86%)	X^2^ = 3.827	0.050
HDP (n, %)	17 (12.06%)	9 (12.68%)	8 (11.43%)	X^2^ = 0.052	0.820
Thyroid dysfunction (n, %)	25 (17.73%)	18 (11.27%)	7 (10.00%)	X^2^ = 5.695	0.017
Hyperlipidaemia (n, %)	13 (9.22%)	11 (15.49%)	2 (2.86%)	X^2^ = 6.724	0.01

Data are presented as numbers (percentages), means ± standard deviations or medians (interquartile ranges). Continuous data were analysed with t tests (for normally distributed data) and expressed as *t* or were analysed with the Mann-Whitney U test (for nonnormally distributed data) and expressed as *Z*. Categorical data were compared using chi-squared tests or Fisher’s exact test and are expressed as *X*^*2*^

### Pregnancy outcomes

The gestational age of the infants in the ultrasound- (21.57–24.86 weeks) and physical examination-indicated cerclage groups (21.82–24.89 weeks) was not significantly different (*P* > 0.05). The mean gestational age at delivery was significantly higher (35.78 ± 4.42 weeks vs. 28.69 ± 5.14 weeks, *P* < 0.001), the cerclage to the delivery interval was subatantially longer (90.76 ± 35.02 days vs. 37.33 ± 32.30 days, *P* < 0.001) and the rate of caesarean Sect. (70.42% vs. 30.00%, *P* < 0.001) was lower in the ultrasound-indicated cerclage group than in the physical examination-indicated cerclage group. The pregnancy outcomes regarding gestational weeks at delivery in the study were divided into five categories: < 28, < 32, < 34, < 37, and ≥ 37 weeks of gestation. The rates of spontaneous preterm birth at < 37, < 34, < 32, and < 28 weeks of gestation were higher in the physical examination-indicated cerclage group (*P* < 0.001). The fetal survival rate (97.18% vs. 75.71%) was higher, the neonatal weight (2785.00 ± 873.73 vs. 1634.34 ± 861.31) was heavier, and the 1-min and 5-min Apgar scores were higher in the ultrasound-indicated cerclage group than the physical examination-indicated cerclage group (*P* < 0.001). There were no significant differences in the rate of complications, such as fever, premature rupture of membranes, cervical laceration, vaginal laceration, perineal laceration, and postpartum haemorrhage between the two groups (*P* > 0.05). The occurrence rate of histologic chorioamnionitis in the physical examination-indicated group (12.68%) was significantly higher than that in the ultrasound-indicated group (35.71%) (*P* < 0.05). The obstetric and neonatal outcomes of the two groups are presented in Table 3.

The study included a total of 15 twin-pregnant women with a mean age of 31.47 ± 3.82 years in the ultrasound-indicated cerclage group and 15 twin-pregnant women with a mean age of 31.47 ± 3.82 years in the physical examination-indicated cerclage group. The age, BMI, and serum parameters of the two groups were not significantly different. In the physical examination-indicated cerclage group, the gestational age at delivery and number of extended days were lower, the preterm birth rate was higher, the birth weight was lighter and the Apgar score (1 min) was lower (details shown in Table 4).

**Table 3:** Comparison of obstetric and neonatal outcomes between the two groups

	Overall (*n* = 141)	Ultrasound-indicated cerclage (*n* = 71)	Physical examination-indicated cerclage (*n* = 70)	Statistic	*P* value
Gestational age at cerclage (weeks)	23.43 (21.71–24.86)	23.43(21.57–24.86)	23.43 (21.82–24.89)	Z = 0.582	0.561
Gestational age at delivery (weeks)	32.26 ± 5.96	35.78 ± 4.42	28.69 ± 5.14	T = 8.788	< 0.001
Extended days (days)	64.23 ± 42.96	90.76 ± 35.02	37.33 ± 32.30	T = 9.42	< 0.001
Mode of delivery				X^2^ = 23.039	< 0.001
Vaginal delivery (n, %)	69 (48.94%)	21 (29.58%)	49 (70.00%)		
Caesarean Section (n, %)	71 (50.35%)	50 (70.42%)	21 (30.00%)		
GA at delivery				X^2^ = 42.801	< 0.001
Full term birth (≥ 37 weeks) (n, %)	53 (37.59%)	44 (61.97%)	9 (12.86%)	X^2^ = 36.244	< 0.001
Preterm birth					
< 37 weeks (n, %)	89 (63.12%)	28 (39.44%)	61 (87.14%)	X^2^ = 34.461	< 0.001
< 34 weeks (n, %)	74 (52.48%)	16 (22.54%)	59 (84.29%)	X^2^ = 53.982	< 0.001
< 32 weeks (n, %)	66 (46.81%)	13 (18.31%)	54 (77.14%)	X^2^ = 48.923	< 0.001
< 28 weeks (n, %)	39 (24.66%)	6 (8.45%)	33 (47.14%)	X^2^ = 26.373	< 0.001
Neonatal outcome				X^2^ = 13.934	< 0.001
Neonatal survival (n, %)	122 (86.52%)	69 (97.18%)	53 (75.71%)		
Neonatal mortality (n, %)	19 (13.48%)	2 (2.82%)	17 (24.29%)		
Birth weight (g)	2285.12 ± 1037.22	2785.00 ± 873.73	1634.34 ± 861.31	T = 7.255	< 0.001
Apgar score (1 min)	10.00 (8.00–10.00)	10.00 (10.00–10.00)	9.00 (6.25–10.00)	Z = 5.434	< 0.001
Apgar score (5 min)	10.00 (9.00–10.00)	10.00 (10.00–10.00)	9.00(7.13–10.00)	Z = 5.339	< 0.001
Neonatal asphyxia (n, %)	5 (3.55%)	3 (4.23%)	2 (2.86%)	-	1.000
Complications					
Fever (n, %)	7 (4.96%)	2 (2.82%)	5 (7.14%)	-	0.275
Premature rupture of membranes (n, %)	39 (27.66%)	19(26.76%)	20 (28.57%)	X^2^ = 0.058	0.810
Cervical laceration (n, %)	32 (22.70%)	16 (22.54%)	16 (22.86%)	X^2^ = 0.002	0.964
Vaginal laceration (n, %)	3 (2.13%)	2 (2.82%)	1 (1.43%)	-	0.568
Perineal laceration (n, %)	19 (13.48%)	11 (15.49%)	8 (11.43%)	X^2^ = 0.499	0.480
Postpartum haemorrhage(n, %)	27 (19.15%)	10 (14.08%)	17 (24.29%)	X^2^ = 0.555	0.456
Chorioamnionitis (n, %)	34(24.11%)	9(12.68%)	25(35.71%)	X^2^ = 10.224	0.001

Data are presented as numbers (percentages), means ± standard deviations or medians (interquartile ranges). Continuous data were analysed with t tests (for normally distributed data) and expressed as *t* or were analysed with the Mann-Whitney U test (for nonnormally distributed data) and expressed as *Z*. Categorical data were compared using chi-squared tests or Fisher’s exact test and are expressed as *X*^*2*^. - Indicates that Fisher’s exact probability method was used, and no chi-square value was output

**Table 4:** Pregnancy outcomes of twin-pregnant women between two groups

	Total number of twin-pregnant women	Ultrasound-indicated cerclage	Physical examination-indicated cerclage	Statistic	*P* value
Patients(n)	30	15	15	-	1.000
Age (years, $\stackrel{-}{x}$ ± s)	30.80 ± 4.15	31.47 ± 3.82	30.13 ± 4.49	T = 0.877	0.388
BMI (kg / m2)	26.03 ± 3.03	26.05 ± 2.86	26.02 ± 3.28	T = 0.031	0.975
ART (n, %)	20 (66.67%)	11 (73.33%)	9 (60.00%)	X^2^ = 0.150	0.699
Serum parameters					
CRP (10^9^ / L)	12.69 ± 12.11	8.60 ± 7.59	16.78 ± 14.50	T = -1.934	0.063
WBCs (10^9^ / L)	10.04 ± 1.81	9.78 ± 2.07	10.31 ± 1.53	T = -0.797	0.432
Neutrophils (10^9^ / L)	9.87 ± 11.64	7.46 ± 1.95	12.29 ± 16.26	T = -1.142	0.263
Lymphocytes (10^9^ / L)	1.66 ± 0.48	1.71 ± 0.49	1.62 ± 0.47	T = 0.513	0.612
Platelets (10^9^ / L)	239.97 ± 68.14	227.87 ± 75.87	252.07 ± 59.56	T = -0.972	0.340
NLR	2.48 ± 2.56	3.12 ± 3.50	1.83 ± 0.70	T = 1.399	0.173
PLR	154.38 ± 58.98	142.63 ± 59.97	166.14 ± 57.56	T = -1.095	0.283
SII (10^9^ / L)	1375.45 ± 990.43	1063.42 ± 541.31	1687.48 ± 1237.07	T = -1.790	0.084
SIRI (10^9^ / L)	3.14 ± 3.10	2.34 ± 1.10	3.95 ± 4.16	T = -1.453	0.157
RTI (n, %)	12 (40.00%)	5 (33.33%)	7 (46.67%)	X^2^ = 0.139	0.709
Gestational age at cerclage (weeks)	23.50 ± 2.46	22.88 ± 2.92	24.11 ± 1.79	T = -1.399	0.173
Gestational age at delivery (weeks)	30.07 ± 4.54	32.96 ± 4.16	27.18 ± 2.73	T = 4.499	< c 0.001
Extended days (days)	35.00 (16.75 ~ 74.50)	71.00 (47.00–93.00)	18.00 (14.00–28.00)	Z = -3.859	< 0.001
GA at delivery					
Full term birth (≥ 37 weeks) (n, %)	4 (13.33%)	4 (26.67%)	0 (0.00%)	-	0.100
Preterm birth					
< 37 weeks (n, %)	26 (86.67%)	11 (73.33%)	15 (100.00%)	X^2^ = 33.725	< 0.001
< 34 weeks (n, %)	22 (73.33%)	7 (46.67%)	15 (100.00%)	X^2^ = 33.484	< 0.001
< 32 weeks (n, %)	20 (66.67%)	5 (33.33%)	15 (100.00%)	X^2^ = 33.597	< 0.001
< 28 weeks (n, %)	11 (36.67%)	2 (13.33%)	9 (60.00%)	X^2^ = 13.966	< 0.001
Neonatal outcome				-	0.483
Neonatal survival (n, %)	28 (93.33%)	15 (100.00%)	13 (86.67%)		
Neonatal mortality (n, %)	2 (0.07%)	0 (0.00%)	2 (13.33%)		
Birth weight (g)	1462.50 (657.50 ~ 2387.50)	2290.00 (1430.00–2605.00)	1020.00 (827.50–1395.00)	Z = -2.971	0.003
Apgar score (1 min)	7.41 ± 2.88	8.43 ± 2.58	6.23 ± 2.84	T = 2.152	0.041
Apgar score (5 min)	8.13 ± 2.14	8.85 ± 2.00	7.31 ± 2.05	T = 2.009	0.055

Data are presented as numbers (percentages), means ± standard deviations or medians (interquartile ranges). Continuous data were analysed with t tests (for normally distributed data) and expressed as *t* or were analysed with the Mann-Whitney U test (for nonnormally distributed data) and expressed as *Z*. Categorical data were compared using chi-squared tests or Fisher’s exact test and are expressed as *X*^*2*^. - Indicates that Fisher’s exact probability method was used, and no chi-square value was output

**Fig. 1:**
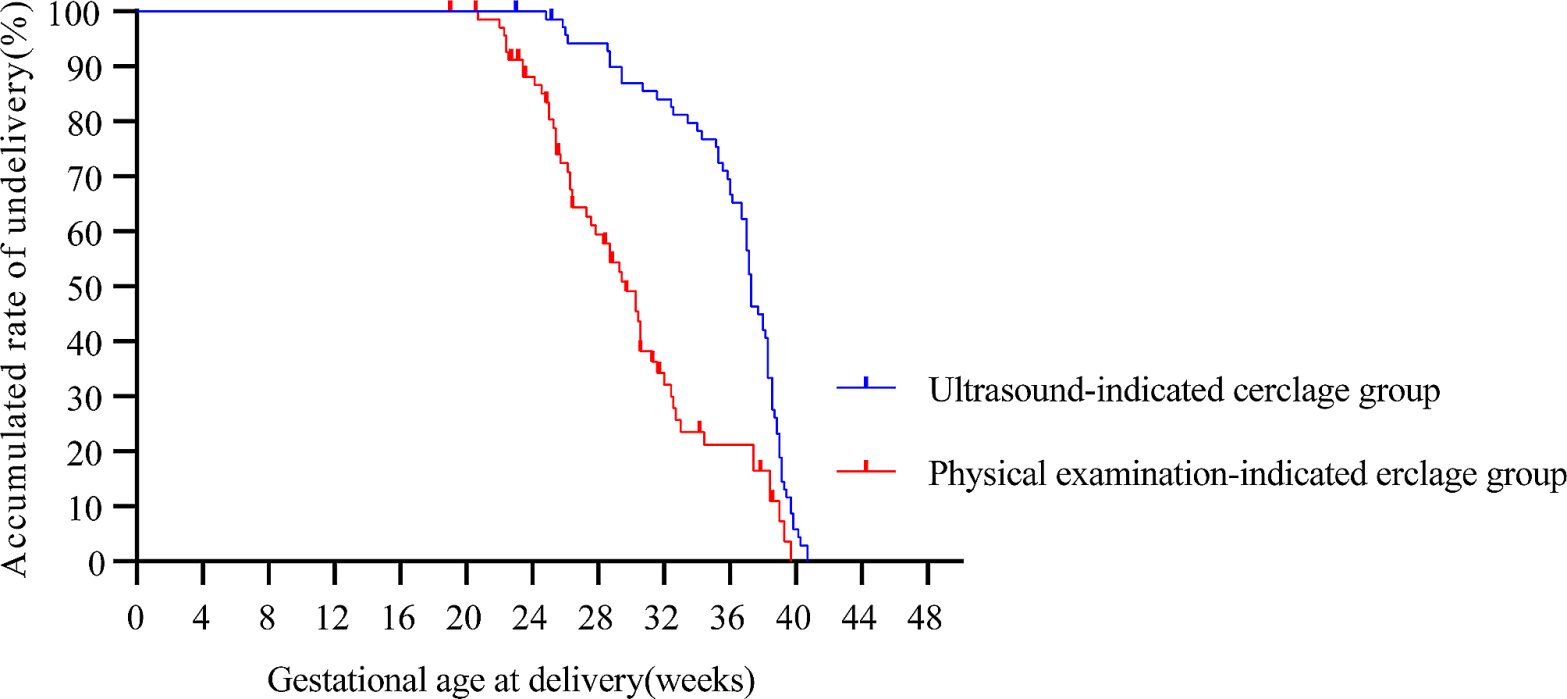
Kaplan-Meier survival curves of gestational age at delivery

Moreover, the survival curves of the two groups were compared based on gestational age at delivery. Kaplan-Meier survival curve analysis showed that the gestational age after cervical cerclage in the ultrasound-indicated cervical cerclage group was higher than that in the physical examination-indicated cervical cerclage group (*P* < 0.05). This finding suggested that women with ultrasound-indicated cervical cerclage might have a better prognosis than those with physical examination-indicated cervical cerclage (37.29 weeks vs. 30.29 weeks in twin-pregnant women, *P* < 0.001) (Fig. 1).

### Factors associated with preterm delivery before 34 weeks of gestation after cerclage

To evaluate the factors that affect the outcome of pregnancy following cervical cerclage, the participants who underwent transvaginal cervical cerclage were divided into two groups according to gestational week at delivery: one group with preterm delivery before 34 weeks of gestation and the other group with preterm delivery at or after 34 weeks of gestation. Multimarker analysis was conducted using logistic regression and ROC analysis to assess the predictability of preterm labor before 34 weeks following cerclage procedure. The proportion of twin-pregnant women, CRP levels, WBC counts, neutrophil counts, NLR, PLR, SII, and SIRI were all significantly higher in the physical examination-indicated cerclage group than in the ultrasound-indicated cerclage group (*P* < 0.05). The results of multivariate logistic regression analysis performed on the influencing factors of gestational age at delivery are shown in Table 5. After adjustment for confounding factors, the results showed that the proportion of twin-pregnant women (OR = 3.829, 95% CI 1.413–10.373; *P* = 0.008), C-reactive protein level (OR = 1.083, 95% CI 1.038–1.131; *P* = 0.022) and SII (OR = 1.001, 95% CI 1.000-1.002; *P* = 0.003) were independent risk factors associated with preterm delivery before 34 weeks of gestation, and twin pregnancy had the highest OR values (Table 6).

**Table 5:** Analysis of factors associated with delivery at < 34 weeks and ≥ 34 weeks of gestation

	Delivery at < 34 weeks of gestation (*n* = 75)	Delivery at ≥ 34 weeks of gestation (*n* = 66)	Statistic	*P* value
Age (years, $\stackrel{-}{x}$± s)	31.08 ± 3.89	31.15 ± 3.66	T = 0.112	0.911
BMI (kg / m^2^)	25.77 ± 3.19	25.18 ± 3.34	T = 1.086	0.279
Singleton/twin pregnancy			X^2^ = 6.209	0.013
Singleton pregnancy (n, %)	53 (70.67%)	58 (87.88%)		
Twin pregnancy (n, %)	22 (29.33%)	8 (12.12%)		
Natural conception/ART			X^2^ = 1.617	0.204
Natural conception (n, %)	28 (37.33%)	47 (71.21%)		
ART (n, %)	18 (24.00%)	48 (72.73%)		
Gravidity (times)	2 (1–3)	2 (1–3)	Z = 0.785	0.432
Parity (times)	0 (0–0)	1 (0–1)	Z = 1.911	0.056
Serum parameters				
CRP (10^9^ / L)	15.25 (6.69–25.12)	8.11 (3.94–12.93)	Z = 3.781	< 0.001
WBCs (10^9^ / L)	10.43 (9.37–11.47)	9.30 (8.05–11.37)	Z = 2.717	0.007
Neutrophils (10^9^ / L)	8.26 (7.27–9.42)	7.09 (6.05–8.85)	Z = 3.324	< 0.001
Lymphocytes (10^9^ / L)	1.53 (1.28–1.88)	1.59 (1.32–1.97)	Z = 0.645	0.519
Platelets (10^9^ / L)	234.28 ± 52.19	217.73 ± 47.63	Z = 1.957	0.052
NLR	5.97 ± 3.43	4.64 ± 1.44	T = 2.947	0.004
PLR	142.70 (124.30–176.23)	128.61 (105.98–154.36)	T = 2.186	0.029
SII (10^9^ / L)	915.1 (1220.13–2050.18)	915.11 (734.94–1128.68)	Z = 4.281	< 0.001
SIRI (10^9^ / L)	2.67 (2.17–3.53)	1.99 (1.47–2.93)	Z = 3.793	< 0.001
RTI (n, %)	27 (36.00%)	16 (24.24%)	X^2^ = 2.290	0.130

Data are presented as numbers (percentages), means ± standard deviations or medians (interquartile ranges). Continuous data were analysed with t tests (for normally distributed data) and expressed as *t* or were analysed with the Mann-Whitney U test (for nonnormally distributed data) and expressed as *Z*. Categorical data were compared using chi-squared tests or Fisher’s exact test and are expressed as *X*^*2*^. - Indicates that Fisher’s exact probability method was used, and no chi-square value was output

**Table 6:** Multivariable analysis of factors associated with preterm birth < 34 weeks of gestation

	β Values		Standard error	*P* value	OR value	95% CI
Twin pregnancy		1.343	0.508	0.008	3.829	1.413–10.373
CRP (109/L)		0.080	0.022	< 0.001	1.083	1.038–1.131
SII		0.001	0.000	0.003	1.001	1.000–1.002

**Fig. 2:**
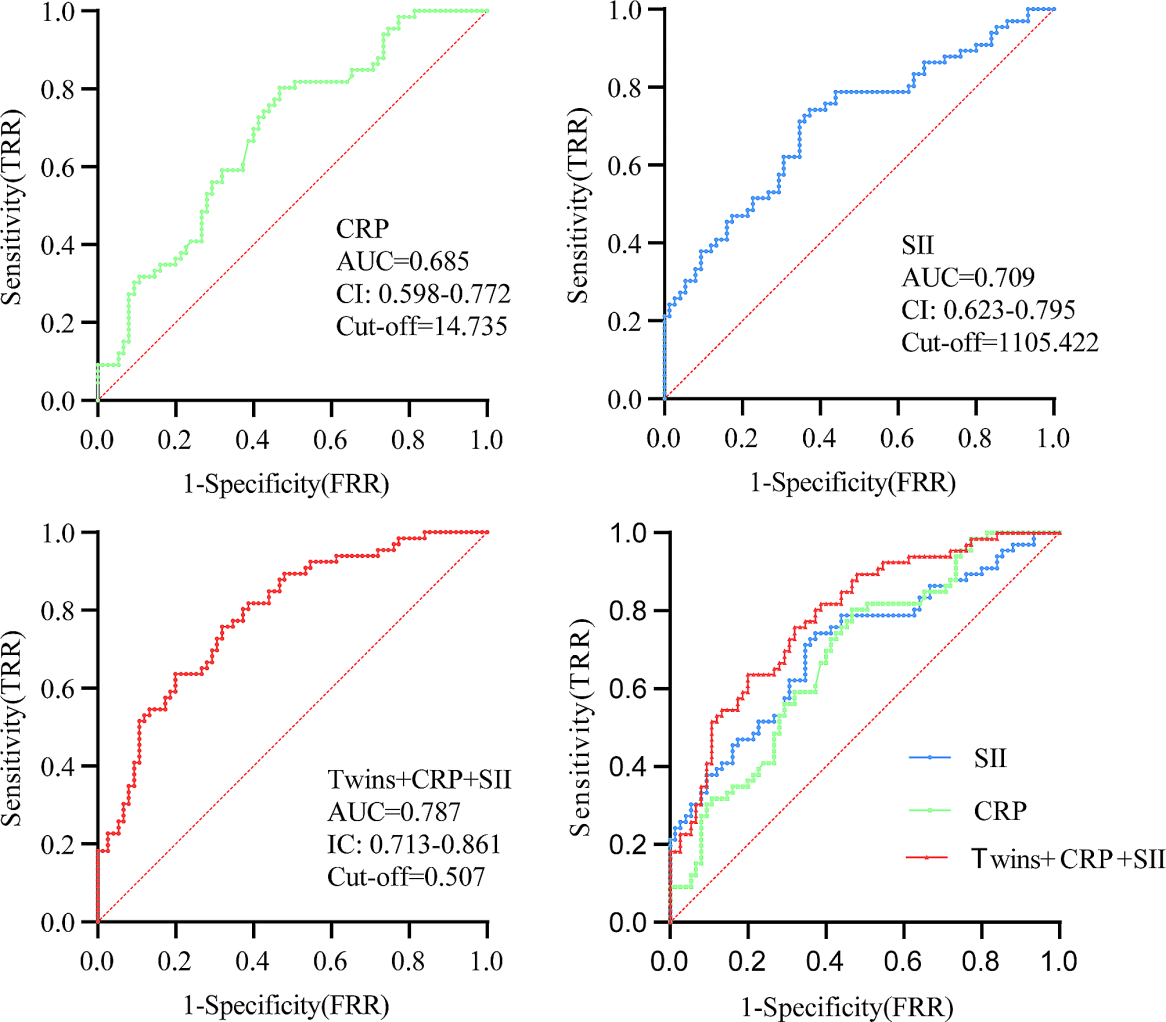
ROC analysis for predicting preterm birth before 34 weeks of gestation

ROC analysis was then performed to determine the predictive value of twin pregnancy, the CRP level, and the SII to predict the outcome of preterm delivery before 34 weeks of gestation following cervical cerclage. The ROC analysis results showed that twin pregnancy had a *P* > 0.05, indicating that twin pregnancy alone could not predict the occurrence of preterm labour before 34 weeks. However, *P* < 0.05 was obtained for the SII and CRP levels by ROC analysis. We found that the AUC of the SII (0.709) was higher than that of the CRP level (0.586). The combination of twin pregnancy, the SII, and the CRP level had a higher AUC (0.787) than either alone. In addition, the optimal cut-off for the SII was 1105.422, and the optimal cut-off for the CRP level was 14.735. The combination of twin pregnancy, the CRP level and the SII had a better performance for predicting the outcome of preterm birth before 34 weeks of gestation after cervical cerclage (Fig. 2).

## Discussion

Cervical incompetence is a well-known risk factor for second-trimester abortion and preterm birth, accounting for approximately 10–25% of recurrent abortions in the second trimester and 40–50% of spontaneous preterm births [12], and remains a significant public health challenge globally. The exact pathogenesis of CIC is unknown and is probably multifactorial. Congenital factors include cervical collagen and elastin deficiencies, Mullerian tube malformation, or intrauterine exposure to diethylstilbestrol, while acquired factors include multiple repeated mechanical dilation of the cervix, cervical conization and loop electrosurgical excision procedures (LEEPs), and obstetric lacerations during induced or emergency labour [13]. Although the etiology of cervical insufficiency is unclear, the last common pathway in the series of events leading to miscarriage or preterm labor is cervical os dilation and uterine extension; therefore, cervical cerclage is a major treatment method to prolong gestation [4].

Gestational age is the main factor affecting the prognosis of newborns, and the morbidity and mortality of preterm infants are closely related to gestational age. Fetuses born at less than 28 weeks of gestation have poorer neonatal outcomes, and their mortality and morbidity are significantly higher [14]. Cervical cerclage can increase gestational age, especially the critical gestational age for newborn survival, improving pregnancy outcomes. In a meta-analysis including ten studies with 757 women, Ehsanipoor et al. reported that cerclage was associated with a significant increase in prolongation of pregnancy for approximately one month and neonatal survival when compared to no cerclage [15]. Similarly, in a systematic review of 15 published trials, Alfirevic et al. reported that pregnant women who underwent cerclage procedures were less likely to give birth before 28, 34, and 37 weeks of gestation than those who received expectant treatment [16]. The pregnancy outcomes of our study mainly included full-term delivery, preterm delivery and late abortion and were further subdivided into groups on the basis of gestational age (< 28 weeks of gestation, < 32 weeks of gestation, < 34 weeks of gestation and < 37 weeks of gestation) [17]. In our study, 72.34% of the patients delivered at or after 28 weeks of gestation, and the neonatal survival rate was 86.52% (122/141). We found that ultrasound-indicated cervical cerclage increased the mean gestational age by 90.76 ± 35.02 days, while cervical cerclage indicated by physical examination increased the mean gestational age by 37.33 ± 32.30 days. Compared with the ultrasound–indicated cerclage group, the physical examination-indicated cerclage group had a shorter interval from cerclage to delivery, a significantly increased risk of preterm delivery at < 28 weeks, < 32 weeks, < 34 weeks, and < 37 weeks of gestation (8.45% vs. 47.14%; 18.31% vs. 77.14%; 22.54% vs. 84.29%; 39.44% vs. 87.14%, respectively; *P* < 0.001), and a higher rate of pregnancy complications such as histologic chorioamnionitis (12.68% vs. 35.71%). Moreover, newborn outcomes, including neonatal mortality, birth weight, and Apgar scores, were poorer in the physical examination-indicated cerclage group. Roman et al. suggested that transvaginal ultrasound surveillance of the cervical length has proven to be a valuable tool for identifying and monitoring preterm birth [18], independent of other risk factors [19].

Although an increasing number of studies have focused on pregnancy outcomes and related risk factors for cervical cerclage with different indications, there is still no definitive conclusion. Contrary to our research, Schaible et al. conducted a retrospective review of 43 patients who underwent transvaginal cervical cerclage and reported no differences in either maternal or fetal outcomes were seen between the physical examination- and ultrasound-indicated groups [5]. Gluck et al. thought that pregnancy outcomes of women with emergency cerclage were comparable with those of women with elective cerclage [20]. However, many studies have provided evidence indicating the superiority of ultrasound-indicated cerclage to physical examination-indicated cerclage in terms of pregnancy and neonatal outcomes. Golbasi et al. evaluated the effectiveness of cerclage according to indications and found that patients who underwent physical examination-indicated cerclage had a significantly higher rate of preterm delivery at < 28 weeks and < 34 weeks of gestation than those who underwent ultrasound-indicated cerclage [21]. Furthermore, Chan et al. considered that patients with physical examination-indicated cerclage had a higher incidence of miscarriage (44.44% vs. 20.0%) and shorter prolongation of gestation (5.7 weeks vs. 17.0 weeks) than those with ultrasound-indicated cerclage [22]. In our study, the patients who underwent physical examination-indicated cerclage had higher risks for adverse pregnancy and fetal outcomes than those who underwent ultrasound-indicated cerclage among women with both singleton and twin pregnancies. Above all, a short cervix is one of the reasons for preterm birth. Patients in the physical examination-indicated cerclage group had a shorter cervical length at cerclage accompanied by cervical dilation, which may be related to poor pregnancy outcomes. Moreover, the majority of patients in the physical examination group with advanced dilatation and membrane bulging through the external cervical os, which directly comes into contact with vaginal flora, are more likely to have infection, and infection is a significant risk factor leading to preterm birth [23].

There is still controversy over whether prolonged pregnancy can compensate for the adverse effects of physical examination-indicated cerclage and ultimately lead to improved neonatal outcomes [24]. Park et al. indicated that intra-amniotic infection plays a vital role in the poor pregnancy outcomes of women with cervical incompetence who undergo physical examination-indicated cerclage [25]. The incidence of intra-amniotic infection and inflammation in patients with mid-trimester cervical incompetence is as high as 50% [26]. Serum inflammatory markers such as CRP and WBC values are recognized as predictive factors for subclinical chorioamnionitis [27]. In this study, the CRP level, neutrophil count, NLR, PLR, SII, and SIRI in the physical examination-indicated cerclage group were higher than those in the ultrasound indication group. This may be associated with subclinical inflammation and infection. We also analyzed the relationship between maternal serum inflammatory biomarkers and pregnancy outcomes after cervical cerclage surgery. The probability of intrauterine infection in physical examination-indicated cerclage patients is significantly higher than that in ultrasound-indicated cerclage patients, leading to poor postoperative prognosis for women with cervical incompetence and inducing preterm birth and other adverse pregnancy outcomes. Moreover, the CRP level, WBC count, neutrophil count, NLR, PLR, SII, and SIRI of the group with preterm delivery before 34 weeks of gestation were higher than those of the group with delivery at or after 34 weeks. Multivariate analysis was conducted to determine that twin pregnancy, CRP level and SII level were found to be independent risk factors for preterm delivery before 34 weeks of gestation following cervical cerclage. Meanwhile the combination of twin pregnancy, CRP level, and SII level had a higher AUC (0.787) in ROC analysis. Moreover, in this study, a total of 33 pregnant women were diagnosed with histologic chorioamnionitis, and they all had preterm delivery before 34 weeks of gestation. In a recent study, Pan et al. included 374 patients who underwent cervical cerclage surgery, of whom 268 (71.7%) had successful surgery, and compared the maternal inflammatory markers of the success and failure groups. They concluded that SII and SIRI in maternal peripheral blood emerged as important biochemical markers for predicting the maternal-neonatal outcome after cervical cerclage [28]. Therefore, maternal peripheral blood levels of inflammatory markers such as CRP and SII should be monitored during the perioperative period to predict outcomes after noninvasive procedures [28] and attention should be paid to monitoring the infection status after cervical cerclage procedure.

However, if cervical dilation is unavoidable, then physical examination-indicated cerclage still has certain benefits for improving perinatal and neonatal outcomes [29]. Extensive research evidence has demonstrated promising outcomes of physical examination-indicated cerclage. Pereira et al. evaluated 225 patients with cervical dilation, of whom 152 underwent physical examination-indicated cerclage, and 73 received expected treatment. The results indicated that the placement of a physical examination-indicated cerclage resulted in a favourable prolongation of pregnancy by ten weeks and a lower rate of preterm birth before 28 weeks of gestation compared with expectant management [30]. Abu et al. reported that physical examination-indicated cerclage can prolong pregnancy by an average of 4–5 weeks, with a 2 - fold reduction in the chance of preterm birth at < 34 weeks of gestation [26]. Chen et al. reported that the duration of pregnancy prolongation was 15.0 (5.0–27.0) days and that the neonatal survival rate was 40.0% in the physical examination-indicated cerclage group [31]. Cockwell et al. reviewed 25 studies and found that the average extended time duration from cerclage to delivery was 7 weeks, and the average fetal survival rate was over 70% [32]. In this study, the mean duration from cerclage to delivery was increased by 37.33 ± 32.30 days, and the survival rate of newborns was 75.71% after physical examination-indicated cerclage.

With the development of assisted reproductive technology, the incidence of multiple pregnancies has increased. Twin pregnancy has a 50% rate of preterm birth [33] and a 12 - fold increased risk of preterm birth compared with singleton pregnancy [34], partly due to cervical incompetence [35]. The incidence of CIC in multiple pregnancy is 5%, which is significantly higher than that in singleton pregnancies, ranging from 0.05% ~ 1.8% [36]. Currently, whether CC should be performed in women with cervical insufficiency who are pregnant with twins or multiples is controversial. Multiple studies have shown that CC does not increase the incidence of preterm labour in twin-pregnant women compared to singleton-pregnant women. In a randomized controlled trial involving 50 twin-pregnant women, whether women with or without a history of PTB underwent cervical cerclage was compared, and cerclage did not seem to reduce the incidence of preterm labor [37]. Roman et al. retrospectively compared 76 cases of twin-pregnant women with cervical dilation ≥ 1 cm who underwent physical examination-indicated cervical cerclage (*n* = 38) and conservative treatment (*n* = 38). Their results showed significant differences in the prolongation of gestation (10.46 ± 5.6 weeks in the cerclage group and 3.7 ± 3.2 weeks in the control group) and neonatal mortality (27.6% in the cerclage group and 59.2% in the control group), reducing the incidence of preterm birth at any given gestational week and improving perinatal outcomes [38]. Barbosa et al. thought that cervical cerclage in twin pregnancy may prolong the pregnancy period, even when placed on a very short or dilated cervix [39]. However, some studies have shown that there is no difference in clinical efficacy between cervical cerclage and conservative treatment for cervical incompetence in twin-pregnant women. A meta-analysis showed that the cerclage group of twin-pregnant women had a significantly higher incidence of preterm birth before 35 weeks of gestation and a trend towards higher preterm mortality rates [40]. Similarly, the Royal College of Obstetricians and Gynaecologists (RCOG) has suggested that cervical cerclage is not routinely recommended in the prevention of preterm labour in women with multiple pregnancies without additional risk factors [7]. Additionally, Berghella evaluated the efficacy of cerclage in 49 asymptomatic twin-pregnant women with a short cervical length of less than 25 mm, with 24 women in the cerclage group and 25 women who did not receive cerclage in the control group; this study showed that cerclage was not associated with the prevention of preterm birth compared with no cerclage and concluded that cerclage cannot currently be recommended for clinical use in twin-pregnant women [41]. The current literature data on the results of emergency cerclage in twin-pregnant women with cervical insufficiency show an average neonatal survival rate of 69.7% (range 50 − 83.8%) [34]. The results of previous studies reported that the mean (min - max) GA at delivery was 27.3(21–34) weeks, and the median latency period from cerclage to delivery was 6.4 weeks [42]. In our research, the live birth rates of twins in women with cerclage were 93.33%, 100% in the ultrasound indication group, and 86.67% in the physical examination indication group. The mean GA of twins at delivery was 30.07 ± 4.54 weeks, and the median cerclage-delivery interval was five weeks. Twin pregnancy was the strongest potent risk factor for subsequent preterm births before 34 weeks of gestation following both ultrasound- and physical examination-indicated cerclage. At present, the evidence on the necessity of physical examination-indicated cerclage surgery in twin-pregnant women is scattered, and further clinical research is needed to explore the relationship between twin pregnancy and preterm delivery.

The study has some limitations. Firstly, the patients who did not give birth at our hospital were not included in this single-centre study, which may lead to some bias in the results. Secondly, this was a retrospective study, so temporality could not be determined. Finally, confounding variables were not taken into account regarding the outcome, including physical exercise and maternal drug treatment. Therefore, prospective studies and randomized controlled trials are essential in the future.

## Conclusion

In summary, we compared the maternal and perinatal outcomes of cervical cerclage based on indications and found that women with ultrasound-indicated cerclage had better pregnancy outcomes than those with physical examination-indicated cerclage. We advocated for the cervical length of transvaginal ultrasound surveillance of CL for pregnant women with risk factors such as a history of prior preterm birth. Twin pregnancy, an elevated CRP level and an elevated SII value were important combined markers for predicting the outcome of preterm birth before 34 weeks of gestation after cervical cerclage.

## Data Availability

No datasets were generated or analysed during the current study.

## Abbreviations

CRP: C-reactive protein
SII: Systemic immune-inflammation index
SIRI: Systemic inflammation response index
PLR: Platelet to lymphocyte ratio
PTB: Preterm birth
CIC: Cervical incompetence
CC: Cervical cerclage
GA: Gestational age
NLR: Neutrophil-lymphocyte ratio
CL: Cervical length
BMI: Body mass index
RM: Recurrent miscarriage
RPL: Recurrent pregnancy loss
RTI: Reproductive tract infection
GDM: Gestational diabetes mellitus
HDP: Hypertension disorders of pregnancy
SPSS: Statistical Package for the Social Sciences
ROC: Receiver operating characteristic curve
OR: Odds ratio
CI: Confidence interval
ART: Assisted reproduction technology
AUC: Area under the curve
LEEP: Loop electrosurgical excision procedure

## References

[1] Vogel JP, Chawanpaiboon S, Moller AB (2018). The global epidemiology of preterm birth[J]. Best Pract Res Clin Obstet Gynaecol.

[2] da Fonseca EB, Damião R, Moreira DA (2020). Preterm birth prevention[J]. Best Pract Res Clin Obstet Gynaecol.

[3] Goldenberg RL, Culhane JF, Iams JD (2008). Epidemiology and causes of preterm birth[J]. Lancet.

[4] Cai S, Wu Y, Zeng L (2022). Effects of vaginal microecology and immunity on the pregnancy outcome of cervical cerclage[J]. BMC Womens Health.

[5] Schaible B, Langhals D, Taylor L (2023). Residency experience with physical examination- and ultrasound-indicated cerclage: a single Center Retrospective Study[J]. Ochsner J.

[6] Hessami K, Kyvernitakis I, Cozzolino M (2022). McDonald versus Shirodkar cervical cerclage for prevention of preterm birth: a systematic review and meta-analysis of pregnancy outcomes[J]. J Matern Fetal Neonatal Med.

[7] Shennan AH, Story L, Cervical, Cerclage (2022). Green-top Guideline 75[J]. BJOG.

[8] Ikechebelu JI, Dim CC, Okpala BC et al. Comparison of Pregnancy Outcomes of History-Indicated and Ultrasound-Indicated Cervical Cerclage: A Retrospective Cohort Study[J]. Biomed Res Int, 2023, 2023: 8782854. 10.1155/2023/8782854 PMC9842428 36654867

[9] Giouleka S, Boureka E, Tsakiridis I (2023). Cervical cerclage: a Comprehensive Review of Major guidelines. Obstet Gynecol Surv.

[10] Hulshoff CC, Bosgraaf RP, Spaanderman MEA, Inthout J, Scholten RR, Van Drongelen J (2023). The efficacy of emergency cervical cerclage in singleton and twin pregnancies: a systematic review with meta-analysis. Am J Obstet Gynecol MFM.

[11] Wang S, Pan X, Jia B, Chen S (2023). Exploring the correlation between the systemic Immune inflammation index (SII), systemic inflammatory response index (SIRI), and type 2 Diabetic Retinopathy. Diabetes Metab Syndr Obes.

[12] Xiao Y, Huang S, Yu W (2023). Effects of emergency/nonemergency cervical cerclage on the vaginal microbiome of pregnant women with cervical incompetence[J]. Front Cell Infect Microbiol.

[13] ACOG Practice Bulletin No (2014). 142: Cerclage for the management of cervical insufficiency[J]. Obstet Gynecol.

[14] Ji X, Wu C, Chen M (2022). Analysis of risk factors related to extremely and very preterm birth: a retrospective study[J]. BMC Pregnancy Childbirth.

[15] Ehsanipoor RM, Seligman NS, Saccone G (2015). Physical examination-indicated cerclage: a systematic review and Meta-analysis[J]. Obstet Gynecol.

[16] Alfirevic Z, Stampalija T, Medley N (2017). Cervical stitch (cerclage) for preventing preterm birth in singleton pregnancy[J]. Cochrane Database Syst Rev.

[17] Thébaud B, Goss KN, Laughon M (2019). Bronchopulmonary dysplasia[J]. Nat Rev Dis Primers.

[18] Drassinower D, Vink J, Pessel C (2015). Effect of cervical cerclage on rate of cervical shortening[J]. Ultrasound Obstet Gynecol.

[19] Roman A, Ramirez A, Fox NS (2022). Prevention of preterm birth in twin pregnancies[J]. Am J Obstet Gynecol MFM.

[20] Gluck O, Mizrachi Y, Ginath S (2017). Obstetrical outcomes of emergency compared with elective cervical cerclage[J]. J Matern Fetal Neonatal Med.

[21] Golbasi C, Golbasi H, Bayraktar B (2022). Effectiveness and perinatal outcomes of history-indicated, ultrasound-indicated and physical examination-indicated cerclage: a retrospective study[J]. BMC Pregnancy Childbirth.

[22] Chan LL, Leung TW, Lo TK (2015). Indications for and pregnancy outcomes of cervical cerclage: 11-year comparison of patients undergoing history-indicated, ultrasound-indicated, or rescue cerclage[J]. Hong Kong Med J.

[23] Huang G, Deng C, Liao H (2022). Comparison of transvaginal cervical cerclage versus laparoscopic abdominal cervical cerclage in cervical insufficiency: a retrospective study from a single centre[J]. BMC Pregnancy Childbirth.

[24] Kuon RJ, Hudalla H, Seitz C (2015). Impaired neonatal outcome after emergency Cerclage adds controversy to prolongation of Pregnancy[J]. PLoS ONE.

[25] Lee J, Lee JE, Choi JW (2020). Proteomic Analysis of Amniotic Fluid Proteins for Predicting the Outcome of Emergency Cerclage in women with cervical Insufficiency[J]. Reprod Sci.

[26] Mönckeberg M, Valdés R, Kusanovic JP (2019). Patients with acute cervical insufficiency without intra-amniotic infection/inflammation treated with cerclage have a good prognosis[J]. J Perinat Med.

[27] Wierzchowska-Opoka M, Kimber-Trojnar Ż, Leszczyńska-Gorzelak B. Emergency cervical Cerclage[J]. J Clin Med, 2021, 10(6). 10.3390/jcm10061270 PMC8003203 33803886

[28] Fang J, Lin Y, Chen Z (2023). The Association of Inflammatory Markers with maternal-neonatal outcome after cervical Cerclage[J]. J Inflamm Res.

[29] Abu Hashim H, Al-Inany H, Kilani Z (2014). A review of the contemporary evidence on rescue cervical cerclage[J]. Int J Gynaecol Obstet.

[30] Pereira L, Cotter A, Gómez R (2007). Expectant management compared with physical examination-indicated cerclage (EM-PEC) in selected women with a dilated cervix at 14(0/7)-25(6/7) weeks: results from the EM-PEC international cohort study[J]. Am J Obstet Gynecol.

[31] Chen R, Huang X, Li B (2020). Pregnancy outcomes and factors affecting the clinical effects of cervical cerclage when used for different indications: a retrospective study of 326 cases[J]. Taiwan J Obstet Gynecol.

[32] Cockwell HA, Smith GN (2005). Cervical incompetence and the role of emergency cerclage[J]. J Obstet Gynaecol Can.

[33] Huang X, Saravelos SH, Li TC (2019). Cervical cerclage in twin pregnancy[J]. Best Pract Res Clin Obstet Gynaecol.

[34] Ekici H, Okmen F, Saritas DG (2023). Cervical cerclage in twin pregnancies: obstetric and neonatal outcomes[J]. Ir J Med Sci.

[35] Chun SH, Chun J, Lee KY (2018). Effects of emergency cerclage on the neonatal outcomes of preterm twin pregnancies compared to preterm singleton pregnancies: a neonatal focus[J]. PLoS ONE.

[36] Zhou X, Li XX, Ge YM (2022). Effects of vaginal microbiota and cervical cerclage on obstetric outcomes of twin pregnancies with cervical incompetence: a retrospective study[J]. Arch Gynecol Obstet.

[37] Dor J, Shalev J, Mashiach S (1982). Elective cervical suture of twin pregnancies diagnosed ultrasonically in the first trimester following induced ovulation[J]. Gynecol Obstet Invest.

[38] Roman A, Rochelson B, Martinelli P (2016). Cerclage in twin pregnancy with dilated cervix between 16 to 24 weeks of gestation: retrospective cohort study[J]. Am J Obstet Gynecol.

[39] Barbosa M, Bek Helmig R, Hvidman L (2020). Twin pregnancies treated with emergency or ultrasound-indicated cerclage to prevent preterm births[J]. J Matern Fetal Neonatal Med.

[40] Berghella V, Odibo AO, To MS (2005). Cerclage for short cervix on ultrasonography: meta-analysis of trials using individual patient-level data[J]. Obstet Gynecol.

[41] Saccone G, Rust O, Althuisius S (2015). Cerclage for short cervix in twin pregnancies: systematic review and meta-analysis of randomized trials using individual patient-level data[J]. Acta Obstet Gynecol Scand.

[42] Cilingir IU, Sayin C, Sutcu H (2018). Emergency cerclage in twins during mid gestation may have favorable outcomes: results of a retrospective cohort[J]. J Gynecol Obstet Hum Reprod.

